# Editorial: Learning a non-native language in a naturalistic environment: insights from behavioral and neuroimaging research

**DOI:** 10.3389/fpsyg.2015.01009

**Published:** 2015-07-17

**Authors:** Christos Pliatsikas, Vicky Chondrogianni

**Affiliations:** ^1^School of Psychology, University of KentCanterbury, UK; ^2^School of Philosophy, Psychology and Language Sciences, University of EdinburghEdinburgh, UK

**Keywords:** bilingualism, second language acquisition, immersion

Research on bilingualism has boomed in the past two decades. The processes by which a second language is acquired and processed has been investigated via linguistic, psycholinguistic, and neurolinguistic perspectives, focusing not only on second language (L2) acquisition and processing, but also the effects it might have on cognition and brain structure and function (Bialystok et al., 2012). More recent studies have focused on the effects of experience-based factors on L2 acquisition and processing (Dussias and Piñar, 2009); for example, several studies have increasingly focused on how L2 processing is affected by the active and continuous use of L2, or immersion, whether it becomes native-like, and which language domains are particularly affected (Dussias and Sagarra, 2007; Pliatsikas and Marinis, 2013). The present E-Book is a collection of recent studies that demonstrate the effects of immersive L2 learning in lexical, phonological and morpho-syntactic processing, while at the same time discusses the potential effects of immersive non-native acquisition on the structure of the bilingual brain.

Several studies in this E-Book have focused on morpho-syntactic processing by immersed late L2 learners. In an ERP study, Carrasco-Ortiz and Frenck-Mestre (2014) showed that highly proficient L2 learners of French with limited immersion (5–6 months) were native-like in their sensitivity of detecting verbal inflectional errors. This sensitivity was enhanced in the presence of phonological cues to the errors, but was also dependent on the L2 learner's overall proficiency. Further evidence in the domain of morpho-syntax was provided in an ERP experiment by Meulman et al. (2014), who demonstrated that immersed (5 years) late Romance learners of Dutch were native-like in detecting auditorily-presented verb agreement violations in non-finite verbs, but not gender violations. This demonstrated that there might be limits to how native-like L2 processing can be, but these limits are specific to the grammatical construction under investigation.

In two behavioral masked lexical priming experiments and in an ERP study with advanced Spanish and German late L2 learners of English, De Cat et al. (2015) showed that lexically transparent noun-noun compounds (NNCs) such as *moon dust* are processed combinatorially by advanced non-native speakers similarly to native speakers; however, sensitivity to word order violations within the NNCs was modulated by the learners' L1.

In an acceptability judgment task, Parafita Couto et al. (2015) examined the interaction between word order and focus in the context of unaccusative (e.g., *arrive*) and unergative (e.g., *walk*) verbs in Spanish in a group of English late L2 learners of Spanish with extensive naturalistic exposure to L2 input. Immersed late L2 learners accepted different word order patterns depending on the focus context; however, they failed to distinguish between unaccusative and unergative verbs, and the ability to do so was a function of the verb's frequency rather than its categorical classification on the basis of unaccusativity. At the same time, L2 learners were less categorical in their judgments compared to monolingual speakers.

In terms of lexical recognition, in two behavioral experiments, Casaponsa et al. (2014) demonstrated that immersed balanced and unbalanced Spanish-Basque bilinguals were equally efficient in recognizing L2-specific bigrams, suggesting that bilingual immersion can lead to native-like orthographic processing; however, these effects were modulated by the participants' L2 proficiency.

Zinszer et al. (2014) tested Chinese-English bilinguals in China and in the US on a lexical categorization task and examined which L2 learner's language history variables (length of immersion, L2 training, age of L2 onset, and code-switching patterns) and language variables (e.g., native speaker agreement on picture naming) predict performance on this task. The authors reported that words with high name agreement and few alternate names elicited high performance; at the same time, immersion, age of L2 onset and code-switching patterns contributed positively to learners' performance, whereas years of L2 training had a negative impact on task performance.

The effects of exposure to naturalistic L2 input on vocabulary learning were examined in two studies by Dahl and Vulchanova (2014) and by Vulchanova et al. (2015). Dahl and Vulchanova examined whether providing naturalistic L2 exposure within a standard school curriculum influences comprehension of vocabulary in two groups of 6-year-old Norwegian-speaking children. After 8 months of exposure, the group that received naturalistic input to English outside the classroom setting but within the school context outperformed on vocabulary learning the group that was only exposed to English within the classroom setting. This suggests that increased exposure to the L2 can lead to a significant increase in receptive vocabulary at this young age even after a short period.

Vulchanova et al. (2015) examined short- and long-term memory effects of first language (L1) and L2 subtitles on text comprehension and vocabulary learning in two groups of adolescent Norwegian learners of English. Short-term effects of L1 and L2 subtitles on text comprehension were found in both groups. These effects were modulated by vocabulary knowledge in the younger group of L2 learners and by knowledge of grammar in the older L2 group. There were no long-term effects in either group on vocabulary learning as measured through a word definition task and lexical decision task. Participants' extracurricular activities such as reading and writing in the L2, exposure to L2 media and games also emerged as significant predictors of the L2 learners' comprehension abilities.

In terms of phonological processing, Gor (2014) demonstrated that heritage English-Russian speakers (early naturalistic interrupted learners) of high proficiency in Russian, were equally efficient to native speakers of Russian in processing speech in noise. This demonstrated the early benefits of immersed L2 learning, which appear to persevere even when immersion is interrupted.

Although the existing behavioral and ERP literature appears to argue for substantial effects of immersion on bilinguals' performance, its effects on brain structure are proven more difficult to capture and describe. In an opinion article, Sharwood Smith (2014) discusses the issues in combining linguistic, psychological and neuroimaging approaches in the search for a unified theory of bilingual processing. In reviewing the neurolinguistic literature, Stein et al. (2014) argue that the reported structural effects of bilingualism on the gray matter (GM) and white matter (WM) of the brain cannot be safely attributed to the type or amount of L2 immersion, although it appears that immersion is more likely to have an impact on the WM (see also Pliatsikas et al., 2015). The effects of bi-/multilingualism on the GM are further demonstrated in a structural MRI study by Kaiser et al. (2015). In this study, possibly the first of its kind on multilinguals, it is suggested that successive L2 learning leads to more extended changes in GM compared to early simultaneous language learning. This effect persists even in individuals that learn a third language later in life, suggesting that early immersive bilingualism might lead to more effective synaptic connectivity for language learning, which in turn leads to less profound structural changes during late learning of additional languages.

Taken together, the papers in this E-book demonstrate the role and the importance of experienced-based factors, and especially linguistic immersion, for the acquisition and processing of a second or a third language. We hope that this E-book will inspire researchers to pay particular attention to the environmental factors that shape the linguistic experiences of their non-native participants, and to present comprehensive descriptions of their groups' linguistic background, including detailed information about their bi-/multilingual immersion.

## Conflict of interest statement

The authors declare that the research was conducted in the absence of any commercial or financial relationships that could be construed as a potential conflict of interest.
